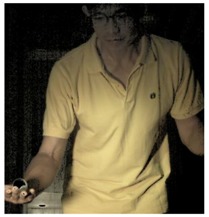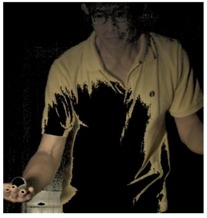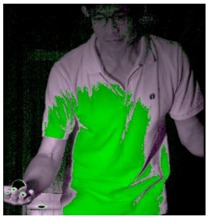# A Study of Automatic and Real-Time Table Tennis Fault Serve Detection System

**DOI:** 10.3390/sports6040158

**Published:** 2018-11-28

**Authors:** Chang-Hung Hung

**Affiliations:** Office of Physical Education, National Chin-Yi University of Technology, Taichung 41170, Taiwan; hongjh@ncut.edu.tw; Tel.: +886-4-23924505

**Keywords:** YCbCr image segmentation, automatic tracking, stable principal component pursuit, computer-aided table tennis ruling

## Abstract

Calling a table tennis fault serve has never been easy for umpires, since they can only rely on their intuition. This study presents an algorithm that is able to automatically find the positions of the ball and racket in the images captured by high-speed camera. The trajectory of ball toss is analyzed and the result can be used as the objective basis for the umpire to decide if the serve is legal. This algorithm mainly consists of YCbCr color space processing, morphological processing method, circle Hough transform application, separation of moving and static components in an image sequence using the stable principal component pursuit method. The experiment results show that YCbCr color space provides better performance than HSV color space in recognizing the ball color close to skin tone. It is also demonstrated that the positions of the ball and racket can be successfully located by using the methods of color segmentation and stable principal component pursuit. Lastly, it is hoped that this study will provide more useful information regarding how to identify illegal ball toss in tennis ball game using image processing techniques to other researchers.

## 1. Foreword

Table tennis is one of the most popular sports in the world. It is also the main sport contested at the Olympic games and Asian games. Many professional table tennis tournaments are held every year. The technical levels are constantly improving, due to the fierce competition among the players. The spin and speed of the ball are enhanced thanks to the continuously improvement on the quality of the rubber and racket. Gaining an upper hand is the important factor to win the match. As the first stroke of the rally, a serve is the key to gaining an upper hand. The importance of an effective serve in the match cannot be overemphasized. In a table tennis game, the server is able to dominate the rally by choosing different speed, spin and landing position of the ball based on his/her tactics, creating an advantage for subsequent attacks. Therefore, each serve can be regarded as the first attack in the rally. A good service gives the player many advantages, such as avoiding the immediate attack from the opponent, creating attack opportunity for the next stroke, etc. The player can even score a point directly with a good service. Therefore, the quality of the serves can have direct influence on the outcome of the game.

In a close game, the result is often decided by the tactics used by the players. With effective service, the server has an advantage of imposing his/her style of play on the opponent, applying tactics and creating opportunity for subsequent attack, and creating favorable conditions for the victory. Therefore, the serve is an important component of exercising his/her tactics in a table tennis game. However, in order to gain the full advantage of by serve, the player may make illegal serve to prevent the opponent from making correct judgment. To maintain the fairness in matches, there strict rules on service and the rules are amended constantly. The rules on service in the International Table Tennis Federation (ITTF) Handbook 2017 [1] are as follows

2.6 The Service
  2.6.1 Service shall start with the ball resting freely on the open palm of the server’s stationary free hand.
  2.6.2 The server shall then project the ball near vertically upwards, without imparting spin, so that it rises at least 16cm after leaving the palm of the free hand and then falls without touching anything before being struck.
  2.6.3 As the ball is falling the server shall strike it so that it touches first his or her court and then touches directly the receiver’s court; in doubles, the ball shall touch successively the right half court of the server and receiver.
  2.6.4 From the start of service until it is struck, the ball shall be above the level of the playing surface and behind the server’s end line, and it shall not be hidden from the receiver by the server or his or her doubles partner or by anything they wear or carry.
  2.6.5 As soon as the ball has been projected, the server’s free arm and hand shall be removed from the space between the ball and the net. The space between the ball and the net is defined by the ball, the net and its indefinite upward extension.

Not every rule regarding illegal serves specifies well-defined reference that a judgment can be based on. For example, in rule 2.6.3 that states, “From the start of service until it is struck, the ball shall be above the level of the playing surface and behind the server’s end line”, the table is a clear reference that the judgment can be based on. According to rule 2.6.2, the height of ball toss shall be at least 16 cm. Rule 2.6.5 states that “the server’s free arm and hand shall be removed from the space between the ball and the net”. These two rules impose certain restrictions on the server and his/her service techniques. Since there is no clear reference, an umpire can only judge if the ball toss height meets the 16 cm requirement and if the ball is blocked by server’s hand based on his/her subjective intuition. The judgment is difficult to make. Without well-defined reference, players are unable to challenge the umpire’s bad calls.

For table tennis, the calls that are argued the most are about the legality of ball toss height and angle. An umpire has to judge if a serve is legal or not promptly based on his/her observation and experience. Many factors can contribute to the misjudgment made by umpires, such as lack of experience, inaccuracy of calls, loss of concentration, etc. The misjudgment may greatly affect the result of the game and the mental conditions of the players.

To ensure fairness in sports games, many image technology based systems such as the Hawk-Eye system, Goal-line technology and Video assistant referee have been used to assist umpires and referees in resolving arguments and making the right calls. The Hawk-Eye system is able to assist the umpires in making accurate judgment in sports games. The Hawk-Eye system, the most popular image technology based umpiring assistance system, has been widely used in many types of sports such as tennis, badminton, volleyball, etc. The Hawk-Eye system has been used in major tennis tournaments, including Grand Slams and all ATP tournaments, for a long time. It has become an integral part of tennis matches.

The rules of legal serves are regularly updated to accommodate the changes in systems and rules. In order to meet the new requirements of the rules, the players and umpires also have to improve their serving skills and umpiring techniques, respectively. Currently, table tennis umpires are making the calls mostly based on their experience and intuitive reaction. Since image technology has not been used to verify the correctness of calls, the players are not given the opportunity to challenge the calls, which is unfair to the players. Researchers have been working on how to assist table tennis umpires in making the right calls by using image technology. It is hoped that the image technology will provide assistance to check the questioned calls. Therefore, the deployment of video technology assisted umpiring system has already become a very important topic for table tennis.

Unlike a line call, which is made based on a clearly identified sideline, making the call of illegal serve in violation of ball toss height requirement is difficult since the umpire has to make the judgment with naked eyes and there is no well-defined reference. The server is always upset with the serve fault call due to the inaccuracy in determining if the ball toss height is over 16 cm. Using video technology to assist umpiring is therefore necessary. This study is motivated by the fact that there are not many available studies on video technology assisted umpiring system for table tennis.

Image technologies have been used to assist in umpiring in many sports, but not yet for table tennis. There are not many studies on using image technologies to assist table tennis umpiring. Zhao et al., propose a model based on the estimation of the trajectory and bounces of the ball. The next step is to obtain the instantaneous information about ball spin [2,3]. Zhang et al., propose an image processing algorithm to locate the current position of a ball and predict its trajectory [4]. Zhao et al., also propose the use of convolutional neural network to find the position and center of mass of a ball [5]. Wong develops an intelligent system which is able to track the location of the ball from live video images and evaluate the service according to the service rules. This system uses artificial neural networks (ANN) to detect the positions of the ball. The performance, however, is affected by the interference from ball-like objects [6]. Wong uses multi-layer perceptron (MLP) and radial basis function network (RBF) at a later time to improve the ball recognition capability. The method only works for still images [7]. The accuracy of ball recognition relies heavily on threshold value, which is very sensitive to noise. Choosing the most appropriate threshold automatically is difficult. Wong and Dooley propose a two-pass thresholding (TPT) method to overcome this sensitivity problem [8]. Myint et al., propose an algorithm to track a ball, in hope of building an automatic umpiring system [9]. Liu et al., present an idea to track the trajectory of an object in real time with stereo camera and to derive the 3D position of the object captured by the camera [10].

In summary, this study is focused on the development of a table tennis umpiring system. The main topics include a variety of image technologies, such as framework design of image processing algorithm, circle (the shape of table tennis ball) location methods, morphological processing methods, color segmentation techniques, etc. The circle Hough transforms is used to locate the circle. This method has advantages of strong anti-noise capability and robust algorithm [11,12,13]. Methods such as average filtering and median filtering are commonly used to remove noise in the image. There are also morphological processing methods, including erosion, dilation, opening, closing, etc. [14,15]. The key lies in color segmentation, i.e., the separation of foreground and background. By using color segmentation technique to separate foreground from background, the computation time can be reduced significantly. However, this technique has to overcome the problem caused by illumination condition [16,17]. The result of color segmentation technique depends on the stability and intensity of illumination. Secondly, the color model chosen will also affect the effectiveness of color segmentation. This issue is mentioned in many studies on skin tone detection. Chaves-González et al. compare the differences among many color models, including RGB (red-green-blue), CMY (cyan-magenta-yellow), YUV (luminance component and two chrominance components), YIQ (luminance in-phase quadrature), YPbPr (green-blue-red), YCbCr (luma-chroma-blue-chroma-red), YCgCr (luminance-chroma-blue-chroma-red), YDbDr (used in the SÉCAM analog terrestrial color television broadcasting standard), HSV (hue-saturation-value), HIS (hue-intensity-saturation), CIE-XYZ (1931 international commission on illumination XYZ color space color models), etc. Of them, HSV delivers the best performance of skin tone detection in this study [18]. Cho et al. propose a skin tone detection method based on adaptive thresholds using HSV color space [19]. Sigal et al. propose a skin color model and its applications using HSV color space [20] as well. It is pointed out in some other studies that YCbCr color space is suitable for skin tone detection [21,22]. Chaudhary conduct experiments under artificial light at night using HSV and YCbCr color spaces, respectively. The results show that YCbCr delivers better performance than HSV [23]. Literature review reveals that each of HSV and YCbCr color spaces are both popular among the researchers. In addition, many researchers are working on how to decompose a given matrix into its low-rank and sparse components [24,25,26]. This method can be used to automatically separate the moving and static components in a sequence of images. In this study, we attempt to apply these techniques to the table tennis umpiring assistance system.

## 2. Research Method

The experiment environment and images related information are described in Section 2.1. The research framework is explained in Section 2.2. Section 2.3 cover the topics of color image processing, including automatic object tracking and racket position detection and how to using stable principal component pursuit algorithm to separate the moving and static components in the images. Section 2.4 deals with the conversion between image pixels and actual dimensions. The goal of this study is to develop a method to find the positions of the ball and racket. The information can be used to assist the umpire in deciding the ball toss curve and ball-striking position for a service. It is also hoped that this study can be used as a reference for other researchers who aim to build a serve fault detection system in the future.

### 2.1. Experiment Environment and Images Related Information

In this experiment, a high-speed camera ix-cameras i-SPEED 210 (F-mount 1280 × 1024 Resolution, 2 μs shutter, 79500 FPS) is used. The camera is placed at a point on the extended end line of the table. The distance between the camera and table is 200 cm. The vertical distance between the camera and ground surface is 100 cm. The high-speed camera captures the images of ball toss at 1000 frames per second and the image size is 880 × 1194 pixels. The subsequent image processing is carried out on each frame. In order not to interfere with the server’s vision, no spotlight is used. The lighting condition is controlled to be as close to the one in an actual match as possible. Therefore, the high-speed shooting is carried under normal illumination. Official table tennis balls of Rio de Janeiro 2016 Olympic Games are used in this experiment.

### 2.2. Algorithm and Processing Framework

**Figure 1:**
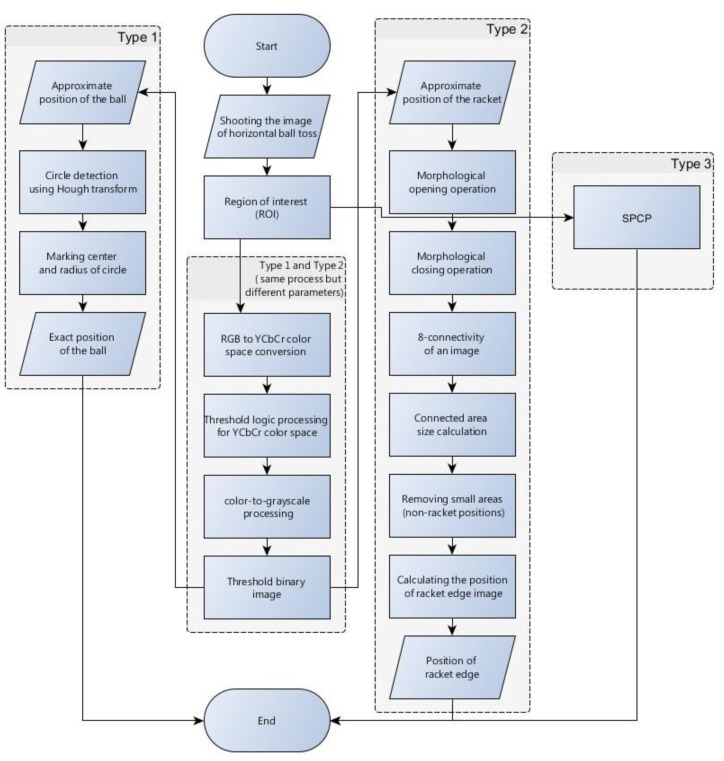
Automatic image processing flowchart.

In this study, the image processing method of horizontal ball toss in an illegal serve is explored. The automatic image processing flowchart is shown in Figure 1. Type 1 and Type 2 represent different steps for automatically locating the positions of the ball and racket, respectively. Type 3 are the steps of using the stable principal component pursuit algorithm to separate the moving and static image components. In this study, a high-speed camera is used to capture the actions of illegal horizontal ball toss by the serving player. The methods proposed in this paper are then tested on the images in order to determine if they are capable of identifying the ball position correctly, assisting the umpire in observing the player’s gesture and making the call correctly. The idea behind Type 1 process is to track the ball and help the umpire observe the ball movement clearly. The goal of Type 2 process is to track the position of the racket so that the umpire can observe the ball and racket when they contact with other easily in order to make the right decision. The Type 3 process allows the umpire to observe the dynamic conditions of the moving ball and racket at the same time. These three methods are also tested and reviewed in the section “Results and Discussion.”

### 2.3. Ball and Background Processing Steps

**Figure 2:**
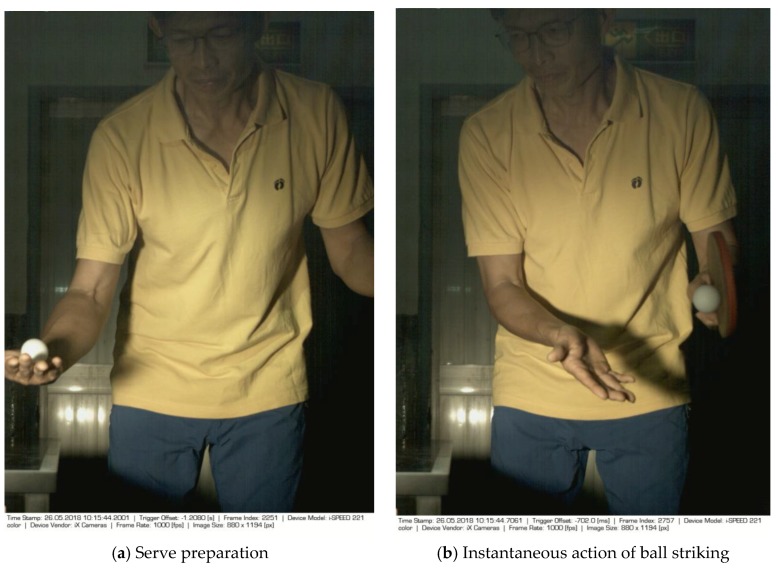
The images of serve preparation and instantaneous action of ball striking.

The images of serve preparation and instantaneous action of ball striking are as shown in Figure 2. The framework of image processing algorithm is shown in Section 2.2. The ROI (region of interest) in each frame is established to remove unnecessary noise, reduce the redundant information and speed up the process to get final judgment. Through Type 1 and Type 2 image processing steps, the approximate positions of the ball and racket can be found. The approximate positions of the ball and racket are separated using YCbCr color space in Type 1 and Type 2 steps, using the same image processing method but different threshold parameters. Table 1 shows the processed results of the critical frames that contain the actions of serve preparation to ball striking. For each critical frame, the original image, ROI image, binarized image and the image of circle finding using the Hough transform are displayed from left to right. The correctness of the ball position identification in the processed image is denoted in the rightmost column. Using the ROI image has the advantage of reducing the amount of data to be processed. Binarization is the color processing technique used to locate the region of the ball. The result of circle finding using the circle Hough transform and the original image are overlapped and compared in order to determine if the correct ball position is found. The associated results of the critical images demonstrate that the proposed processing steps and methods for ball identification are feasible. The results of binarization indicate that the effectiveness of this process is affected by the light source. Two ball-like white shapes are seen in the binarized image of the 240th frame, which may cause misjudgment. Therefore, the condition of the light source is a factor that requires attention.

**Figure 3:**
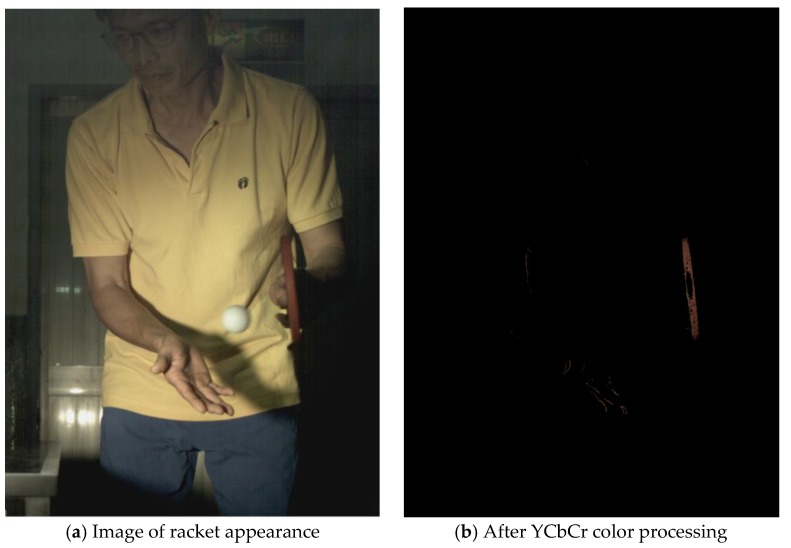
The position of racket after Type 2 YCbCr color processing.

The approximate position of the ball can be located after Type 1 color processing. To further identify exact position, the original image is first turned into a binary image. Then the circle Hough transform is applied to extract the circle. If successful, the information about the center and radius of the circle is recorded. This information gives the exact position of the ball. Table 1 shows the images of some frames and corresponding results. As shown in Figure 3, the approximate position of the racket can be located after Type 2 color processing. To help find more accurate position, morphological opening operation and close operation are first used in this process to reduce the noise. The connected component analysis method is then used to find the position of racket edge. In this method, 8-connected components and their sizes are calculated and non-racket positions are abandoned based on the results. In addition, Type 3 uses the stable principal component pursuit (SPCP) to automatically separate the background and moving images. This concept is applied to the table tennis related research topics in this study. The concept is shown in the equation below [26].

$$\underset{L,S}{\mathrm{minimize}}\;\max\;\left({\left|\|L\|\right|}_{*},\lambda_{\max}{\left\|S\right\|}_{1}\right)$$

$$\mathrm{subject}\;\mathrm{to}\;\|L+S-Y\|{}_{F}\leq \varepsilon ,$$

This method is called “max-SPCP”, where *L* is the low-rank matrix, λ_max_ a positive constant, *S* the sparse matrix, 1-norm ${\left\|\cdot \right\|}_{1}$ and nuclear norm ${\left\|\cdot \right\|}_{*}$, *Y* the noisy matrix and parameter ε the error constant. Given a sequence of images *F*, the solutions found using the above concept and equation indicate that the matrix *L* has a strong correlation with long-time static images. Also, the matrix *S* has a strong correlation with moving object in the images. The changes in pixels are huge but the number of pixels exhibiting changes is small, which matches the characteristics of a sparse matrix. Simply put, continuous image frames can be separated into strongly-correlated background images (i.e., low-rank matrix) and foreground images (i.e., sparse matrix).

### 2.4. The Conversion of Image Pixels and Actual Dimension

**Figure 4:**
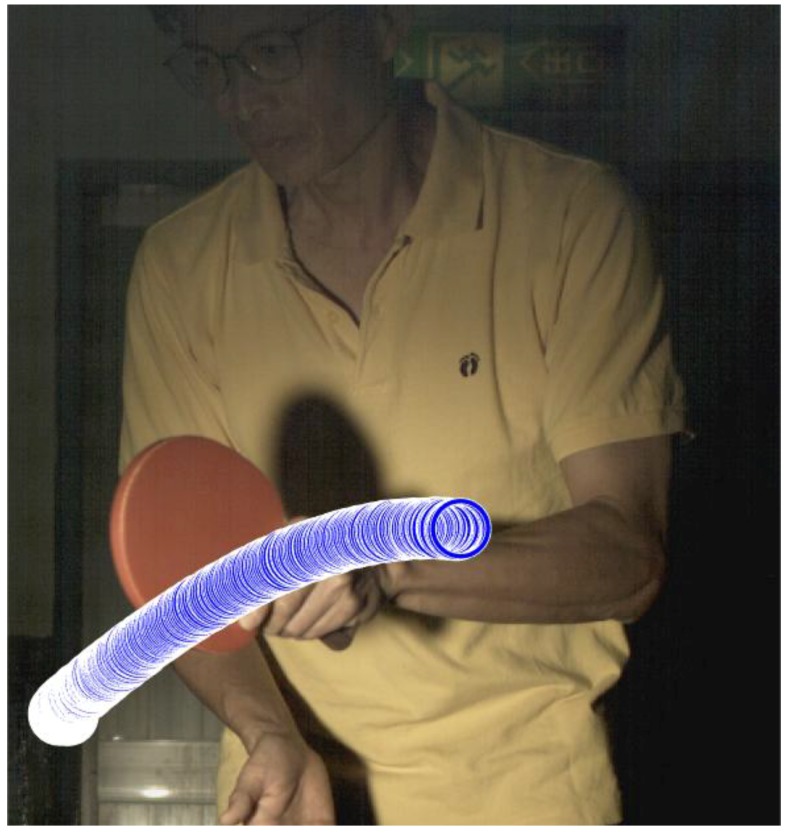
The ball trajectory automatically tracked and recorded (recorded since the ball appears).

In this study, the white end line of table is used as a reference for the conversion between image pixels and actual dimension. The table used in the experiment is Nittaku 3250 model and the width of the end line is 5.5 cm, which is rule compliant. Table 2 shows the coordinates of the two points of white end line in the image are (X, Y) = (83, 1096) and (125, 1096), meaning that the width of white end line is 42 pixels. Therefore, each pixel in the image represents 5.5 cm/42 pixels = 0.13095238095 cm. The server stands very close to the side of the table. The position of the ball is automatically detected by Type 1 algorithm and the trajectory is recorded as shown in Figure 4.

## 3. Results and Discussion

### 3.1. Dynamic Ball and Racket Tracking

A high-speed camera allows us to obtain every important image frame of the moving object. Its high frame rate ensures the player’s action and ball trajectory can be redisplayed clearly. Despite the advantage, the short exposure time of high-speed shooting requires enough light in order for the image sensor to work properly. Therefore, complementary lighting is always needed. In this study, an attempt is made to use Godox LED36 as lighting equipment so that the player will not be affected. Image processing techniques are also used to track the positions of the ball and racket. The corresponding processed results of different frames in Table 1 indicate that the algorithms are effective in achieving the goals in different stages. It is feasible to locate the correct position of the ball automatically in a noisy binary image using Hough transform. The blue trajectory of the moving ball is shown clearly in Figure 4. The trajectory is created from the ball positions in the images. Hough transform delivers excellent performance in locating the circle and exhibits anti-noise capability. The YCbCr color processing technique is used for racket tracking. The results shown in Figure 3 indicate that the method is remarkably effective in color tracking as well. When reviewing the entire image sequence, it is discovered that using method such as color segmentation produces mostly correct results. The color segmentation method fails only in very few cases due to unstable lighting condition. As for racket tracking, the position of racket can be correctly located if the red surface faces directly to the camera.

Figure 4 shows the motion trajectory of the ball is clearly identified and represented in blue color in the images. In this example, the ball is tossed horizontally. Although the ball toss distance is greater than 16 cm, it is still considered a fault serve due to the wrong toss angle. The method proposed in this study is actually capable of tracking the ball and racket. This color tracking based real-time system is useful in assisting the umpire in making the right calls.

The past studies on table tennis umpiring assistance systems are mostly focused on the topics of ball detection and tracking. In this study, YCbCr color space is used to separate the approximate positions of the ball and racket. The morphological opening operation and closing operations are used in the Type 2 process to solve the problem caused by noise in order to find the exact positions of the ball and racket. The exact point of ball striking can be found in this way as well. This method is useful in umpiring based on the rule 2.6.4 “From the start of service until it is struck, the ball shall be above the level of the playing surface and behind the server’s end line”.

### 3.2. The comparison of HSV and YCbCr Color Spaces in This Study

The tracking of ball and racket can be realized by image segmentation process using color space. The position of the ball, once located, can be used as the starting point from which the ball leaves the hand. Although some studies mention that HSV color space is more suitable for skin color identification [18,19,20], the ball, skin and jersey have similar colors in this study. However, YCbCr color space is believed to detect skin color better [21,22,23] in the other group of studies. Table 3 shows how the regions of the ball, jersey and skin are highlighted with image processing techniques using HSV and YCbCr color spaces. The results are compared and the regions with different intensities, depicted in magenta and green, are shown in the rightmost column. Therefore, the experiment carried out in this study will explore and compare the performance of separating the colors of the ball, jersey and skin by using HSV and YCbCr color spaces. The results are shown in Table 3, which indicate that YCbCr is better than HSV in image segmentation for ball and skin recognition, but HSV is better in image segmentation for jersey recognition. Therefore, YCbCr delivers better performance in object tracking than HSV in the environment of this experiment. In addition, YCbCr is slightly better than HSV color space in color segmentation.

The goal of this study is to develop a system that is capable of assisting an umpire in judging an illegal serve. In order to help the umpire making the final decision, the images recorded will be replayed repeatedly and the processed image will be overlaid on the original one. The actual distance from the pixel positions in the image can be obtained based on the relation of actual object size and pixels representing the object.

### 3.3. SPCP Application and Results

**Figure 5:**
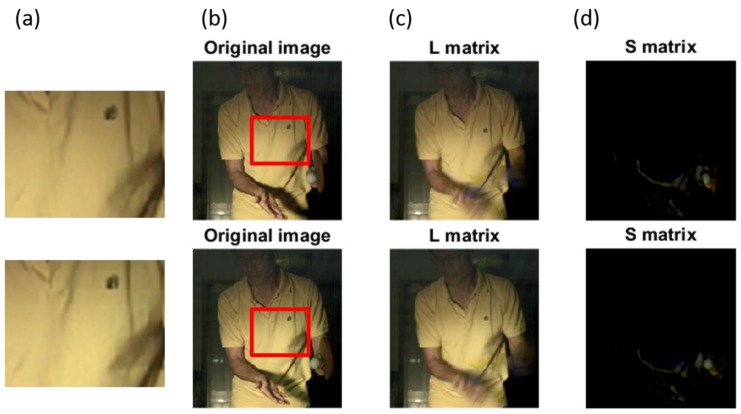
Shows the results of the sequence of images processed with stable principal component pursuit (SPCP): (**a**) The magnified boxes in (**b**), with different brightness, (**b**) two original images, (**c**) matrix L and (**d**) matrix S.

The results of SPCP processing are shown in Figure 5. Complementary lighting is used to provide the light required by high-speed shooting. However, the unstable light source causes the difference of brightness and darkness between the images, as shown in Figure 5a. It also affects the results of ball and racket extraction using color segmentation. Stable moving and static images can be obtained by using SPCP, as shown in Figure 5c,d, respectively. The positions of the ball and racket in the moving images can be easily observed. To assess the performance of color tracking by SPCP, it can be discovered that SPCP performs very well in separating moving and static components. The positions of the ball and racket can also be clearly identified by observing the numerical results of matrix S. However, the SPCP algorithm requires large amount of computation time. Although the SPCP algorithm with quasi-Newton acceleration method [26] is used in this study, the computation time needed in real situation is still much greater than color segmentation method. A certain amount of images is also required in order to find the solution. Using color segmentation method to find solution quickly could lead to wrong solution in unstable lighting condition. It is therefore suggested by this study that color tracking be mainly used if there is a need for real-time processing and SPCP be used as second option. When there is no need for real-time processing, the SPCP is the best choice and color tracking the next.

## 4. Conclusions

It has become a trend to use image technologies as an umpiring aid. To help decide the legality of a ball toss height, this study proposes a method to process images automatically and track the positions of the ball and racket by using images captured by high-speed camera. In the process, the positions of the ball and racket can be quickly located by taking the steps in Type 1 and Type 2, respectively. The experiment results indicate that it is feasible to track the positions of the ball and racket by using YCbCr color segmentation technique. It is also known that YCbCr color space delivers better performance than HSV color space. High-speed camera has the advantage of capturing many frames in a short time. The SPCP method can be used on the images captured by high-speed camera in order to separate the static and moving components in the sequence of images. A fault serve can be decided by observing the accurate motion trajectory of the ball and the frame of instantaneous ball striking action. Lastly, we hope that this study will provide more useful information regarding how to identify illegal ball toss in tennis ball game using image processing techniques to other researchers. The goal of the system to be developed is to improve the umpire’s correctness and to provide a tool with which players can enhance their skills as well.

## Figures and Tables

**Table 1:** The results of image processing of different frames in Type 1.

Frame		The Correctness of the Position of the White Ball (Yes/No)
1	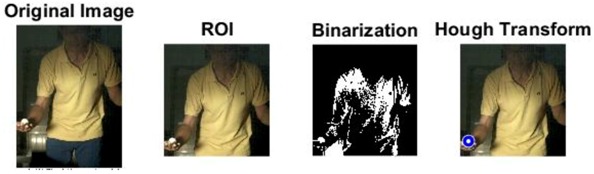	Yes
30	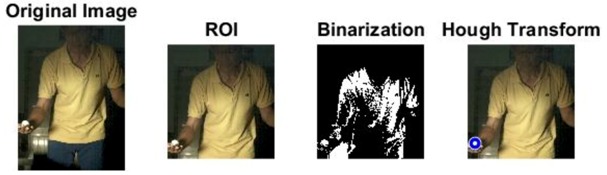	Yes
180	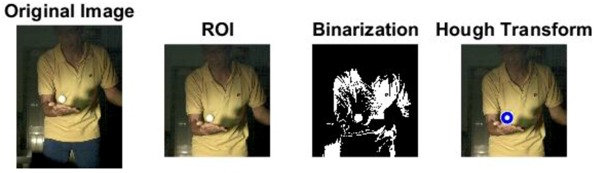	Yes
240	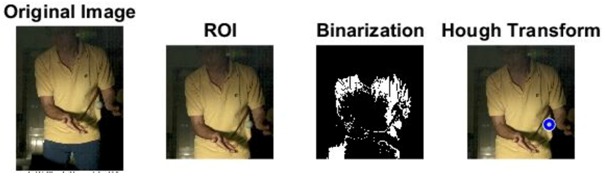	Yes
258	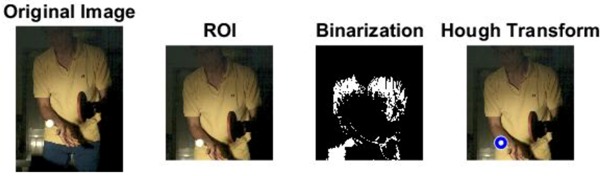	Yes

**Table 2:** The data of actual width and pixel number.

	Actual Width	Pixel Number in the Image	Position in Image
**White line**	5.5 cm	42 pixels	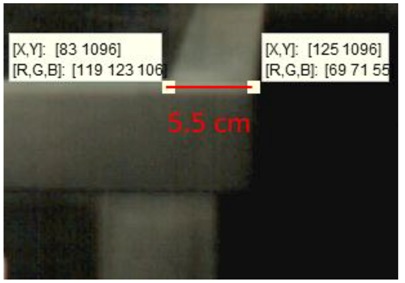

**Table 3:** The results of image segmentation using HSV and YCbCr color spaces.

	HSV	YCbCr	Compare Differences between Images
The ball	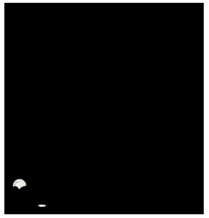	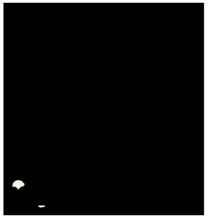	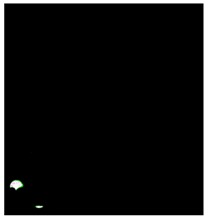
Jersey	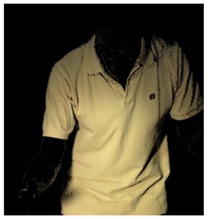	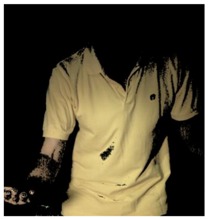	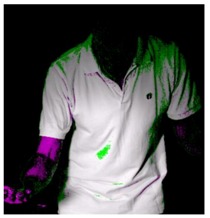
Skin